# Low-Grade Mucoepidermoid Carcinoma Mimicking a Mucocele in the Soft Palate: A Case Report

**DOI:** 10.7759/cureus.103989

**Published:** 2026-02-20

**Authors:** Kenia Yanira Baños-Hernández, Edna Zoraida Rojas-Curiel, Danny Rolando Soria-Cespedes, Francisco Javier Saynes-Marín, Montserrat Ramírez-Rivas

**Affiliations:** 1 Otolaryngology, Hospital Angeles Lindavista, Mexico City, MEX; 2 Pediatric Oncology Surgery, Hospital Angeles Lindavista, Mexico City, MEX; 3 Pathological Anatomy, Hospital Angeles Lindavista, Mexico City, MEX; 4 Otolaryngology, Hospital Angeles Metropolitano, Mexico City, MEX; 5 Medical Education, Hospital Angeles Lindavista, Mexico City, MEX

**Keywords:** minor salivary glands, mucocele, mucoepidermoid carcinoma, pediatrics, soft palate

## Abstract

Mucoepidermoid carcinoma (MEC) is the most common malignant salivary gland tumor, but its occurrence in the soft palate and among pediatric patients is unusual. This case highlights the rarity of this condition in children. A nine-year-old girl with a history of recurrent respiratory infections and allergic rhinitis with nasal obstruction developed a cystic, fluctuating lesion on the right side of her soft palate, initially diagnosed as a mucocele. She underwent septoplasty with complete removal of the lesion, and a low-grade mucoepidermoid carcinoma was incidentally diagnosed. MECs usually present as painless, slow-growing tumors that are often mistaken for benign growths because of their appearance and the presence of many differential diagnoses. This case underscores the importance of histopathological examination of seemingly benign palate lesions and highlights the need for a multidisciplinary approach when an incidental finding is made.

## Introduction

The salivary glands (SGs) include the parotid, submandibular, and sublingual glands, collectively known as the major salivary glands, along with submucosal groups of salivary tissue scattered throughout the oral cavity, paranasal sinuses, pharynx, and upper respiratory tract (called minor salivary glands) [1].

Diseases of the SGs are uncommon in children and adolescents and are usually inflammatory [1,2]. SG neoplasms are rare, accounting for 3%-6% of head and neck tumors. Epithelial tumors are extremely rare, and most tumors in this age group are benign non-epithelial neoplasms [2-4].

The distribution of lesions in the oral cavity of children, in order of frequency, is reported as follows: lips, approximately 22%; tongue, 21%; cheeks, 19%; floor of the mouth, 13%; minor salivary glands, 13%; alveolar ridge, 8%; maxilla, 4%; and palate, 3.3% [5]. The vast majority of oral lesions in children are benign and mesenchymal (84%-99%), although malignant tumors should be considered at any age [5].

A mucocele is a lesion associated with the minor salivary glands of the oral cavity that results from duct rupture and mucin leakage into the surrounding soft tissues [6]. Clinically, it appears as a rounded, slightly bluish mucosal swelling that fluctuates in size, and when located deeper, it may be normochromic. The most common location is the lower lip, followed by the ventral surface of the tongue, the upper lip, the floor of the mouth, and the palate [6]. Mucoceles are among the most frequent lesions in pediatric patients, with a prevalence of 65.68%. Despite their benign nature, they can interfere with feeding and breathing, so proper treatment is necessary [7]. The preferred treatment is conventional surgical excision [6-8].

Conversely, mucoepidermoid carcinoma (MEC) is the most common malignant tumor of the salivary glands [9]. The parotid gland is the most frequent site (48%), followed by minor salivary glands (36%) and the submandibular gland (16%). For minor salivary glands, the palate is the most common site for MEC development, with other locations including the tongue, lips, buccal mucosa, and retromolar region [4,10].

We report the case of a pediatric patient with a lesion on the soft palate, initially diagnosed as a mucocele but later reclassified as mucoepidermoid carcinoma after histopathological analysis.

## Case presentation

**Figure 1:**
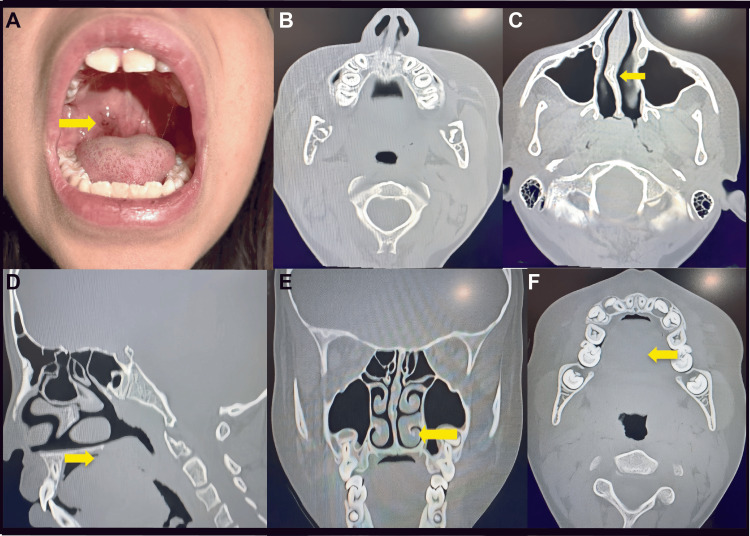
Representative images from the physical examination of our patient and the CT scan of the nose and paranasal sinuses. A. Well-defined, cystic-appearing lesion with fluctuating size during upper respiratory tract infections (URTIs) and their remission (yellow arrow). B. Axial section at the level of the palate that does not show the lesion. C. Axial section showing septal deformity in Cottle's area II to IV block with a contacting spur (yellow arrow). D. Sagittal section that does not show the lesion in the palate. E. Coronal section showing turbinate hypertrophy (yellow arrow). F. Axial section with no evidence of a cystic lesion.

A nine-year-old girl with no family history of cancer presented with a history of allergic rhinitis and recurrent upper respiratory infections causing significant nasal obstruction and mouth breathing. Months later, her parents reported a 1 cm increase in the volume of the right soft palate, which fluctuated in size, enlarged during respiratory infections, and regressed after the infections resolved. Physical examination showed a deviation of the nasal septum to the right, from Cottle areas II to IV, with a contacting spur in area III and turbinate hypertrophy. The oral cavity revealed a lesion with well-defined borders, matching the color of the surrounding mucosa, occasionally erythematous, non-tender to digital pressure, without purulent discharge, and of soft consistency (Figure 1A). The palatine tonsils were grade II and appeared normal. The lesion was aspirated, yielding mucoid material and reducing its size, raising suspicion of a mucocele or retention cyst.

**Figure 2:**
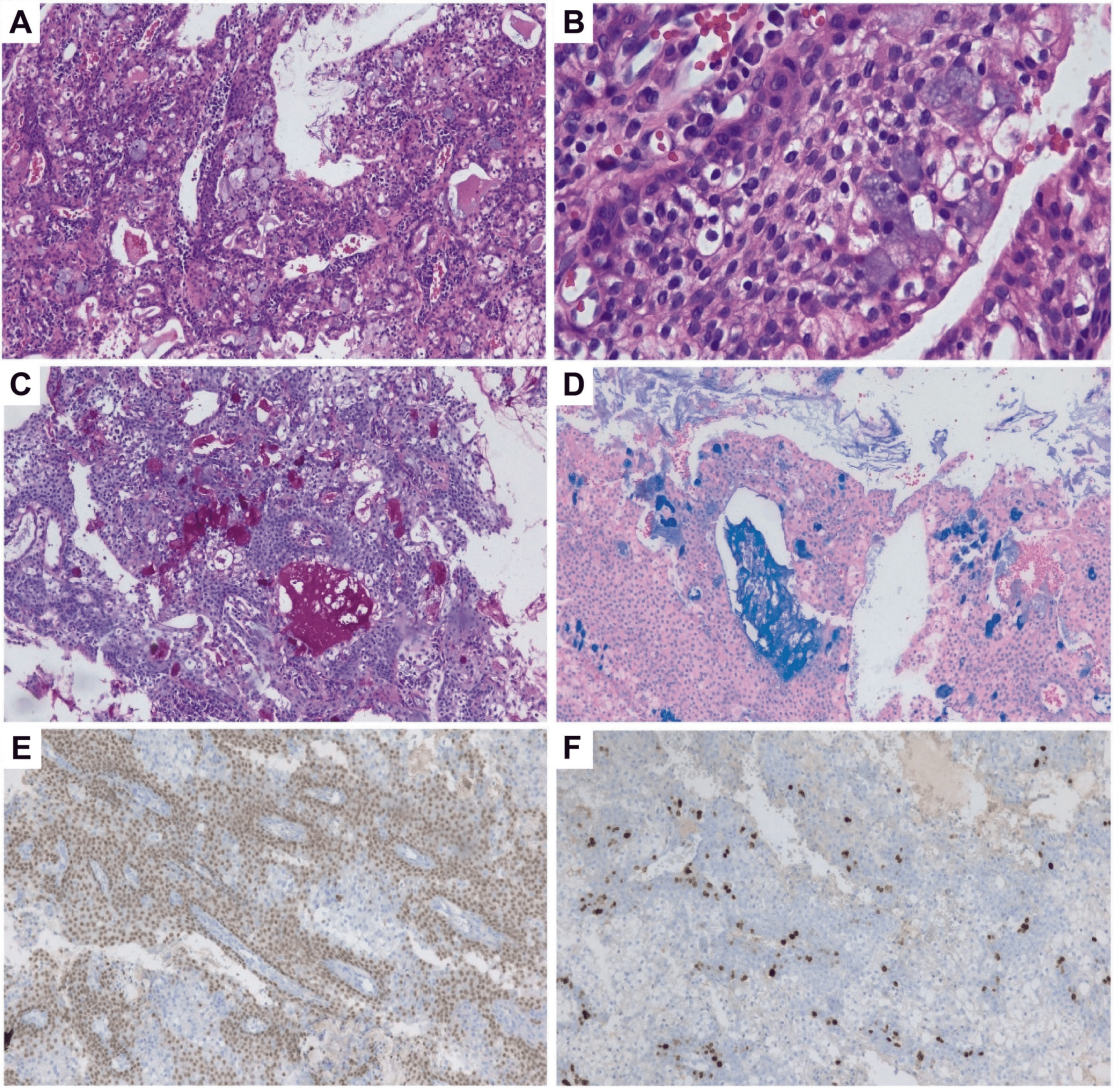
Histopathological analysis of the lesion. A. Histological section of a palatal tumor with cystic space formation, containing squamous, intermediate, mucin-secreting, and clear cells (hematoxylin and eosin (H&E) stain, 100x). B. At closer magnification, the cells have ovoid, regular nuclei with homogeneous chromatin (H&E stain, 400x). C and D. Mucin-secreting cells are identified (100x) in the periodic acid-Schiff (PAS) stain (C) and the Alcian blue stain (D). By immunohistochemistry, the neoplastic cells are p40-positive (E) and have a Ki67 proliferation index of 15% (F) (Immunohistochemistry 100x).

A CT scan of the nose and paranasal sinuses revealed a septal deviation with an obstructive pattern, without clearly visualizing the palatal lesion (Figures 1B-F). The patient was scheduled for functional septoplasty with turbinate remodeling and complete resection of the suspected mucocele. The lesion was sent for histopathological examination, and mucoepidermoid carcinoma was incidentally reported. Based on this finding, immunohistochemical analysis was requested (Figures 2E,F), and the patient was referred to the clinical and surgical oncology service.

The histopathology study reported a malignant epithelial neoplasm composed of various cell types, predominantly cells with abundant, clear cytoplasm and ovoid nuclei, exhibiting slight variation in size and shape, with one mitotic figure per 10 high-power fields at 40x magnification and no necrosis. Squamous cells with abundant eosinophilic cytoplasm, regular ovoid nuclei, homogeneous chromatin, and occasional nucleoli were also present. Mucus-secreting cells with abundant multivacuolated cytoplasm were identified among these cell populations. These cells were positive with Alcian blue and periodic acid-Schiff (PAS) staining with diastase, had small ovoid nuclei, and surrounded cystic spaces containing basophilic mucinous material. Additionally, intermediate cells with scant cytoplasm, ovoid nuclei, and dense chromatin were present in the basal region. Immunohistochemistry showed multifocal p40 positivity (Figure 2E); SOX10 was negative, and Ki67 was positive in 15% of the neoplastic cells (Figure 2F). These morphological and immunostaining characteristics support a diagnosis of low-grade mucoepidermoid carcinoma.

**Figure 3:**
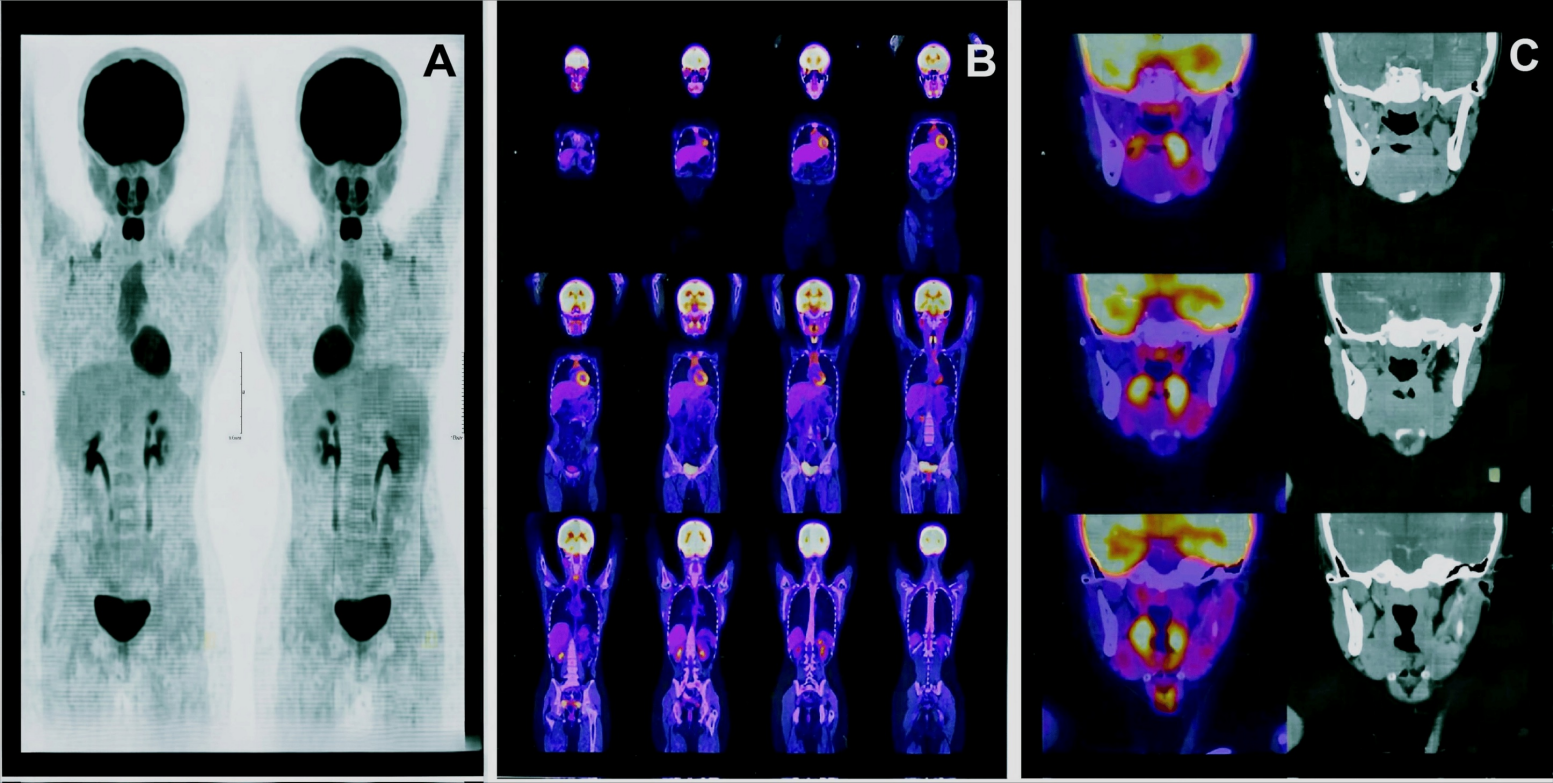
Follow‑up whole‑body and regional PET/CT imaging demonstrating absence of metabolically active disease. Panel A shows paired whole‑body PET maximum‑intensity projections. Panel B presents sequential coronal PET/CT fusion slices from head to pelvis. Panel C displays fused PET/CT and corresponding CT images of the head and neck. The follow‑up PET‑CT scan revealed no pathological metabolic uptake at the surgical bed, within the cervical lymph node chains, or at distant sites, indicating no evidence of metabolically active tumor disease. These findings support a favorable prognosis, for which periodic clinical and imaging surveillance is advised.

A PET-CT scan revealed paranasal sinuses with adequate development and pneumatization, an oral cavity without evidence of lesions, and no focal areas of abnormal hypermetabolism. The nasopharynx, oropharynx, and hypopharynx showed no evidence of hypermetabolic lesions, and the cervical lymph nodes were of normal size and morphology (Figure 3).

**Figure 4:**
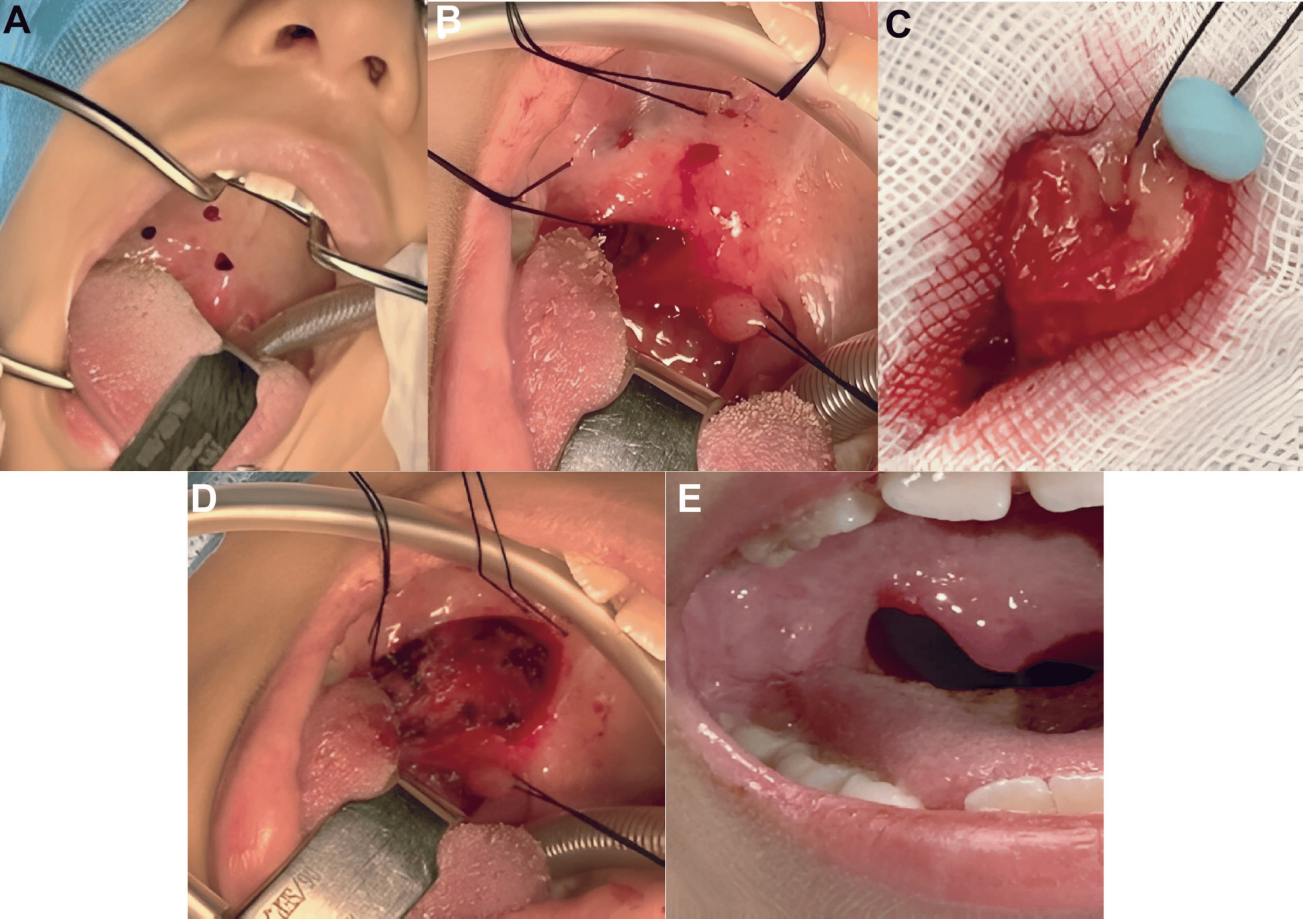
Widening of surgical margins and postoperative follow-up. A and B: Marking of the surgical area. C: Surgical specimen with expanded margins. D: Postoperative surgical bed. E: Postoperative image one month after surgery showing adequate healing by secondary intention.

Based on these findings, the pediatric surgical oncology team performed surgery to widen the surgical margins (Figure 4). A second histopathological examination confirmed that the superior, inferior, internal, and external margins, as well as the surgical bed, were free of neoplastic cells.

The patient is progressing well and is under evaluation by the clinical oncology team, which determined that only close monitoring is necessary, with no need for radiotherapy or chemotherapy. Follow-up will be conducted in a multidisciplinary outpatient setting. Currently, there is no evidence of clinical or radiological recurrence. The follow-up schedule includes visits every 15 days for the first three months, then annually for the next five years, for a total of 10 years.

## Discussion

MEC is an epithelial neoplasm that can originate in the major and minor salivary glands. It accounts for 25% of salivary gland tumors and about 50% of malignant tumors. MECs are rare in children, with approximately 1% to 5% of salivary gland tumors occurring in children and adolescents [3,4,11]. These tumors are more common in individuals between the third and sixth decades of life and are reported more often in women [11,12].

When they occur in the oral cavity, MECs are most common on the palate, with the junction of the hard and soft palate being the most frequent site [12,13]. It is important to consider differential diagnoses with other oral lesions, since these cystic lesions can be mistaken for a mucus retention phenomenon, such as a mucocele, based on their clinical appearance [1,4]. For example, in this patient, the medical history and physical examination suggested a mucocele. The similar appearance of low-grade MECs and mucoceles results from the formation of mucous cysts and pseudocysts. When diagnosing a bluish or mucosa-colored mass that is compressible or fluctuant in an intraoral area containing salivary glands in a child or adolescent, reactive and neoplastic lesions should be considered; mucocele and MEC should be at the top of the differential list [14]. Other possible diagnoses include odontogenic abscesses, mesenchymal neoplasms, pleomorphic adenoma, adenoid cystic carcinoma, adenomatoid hyperplasia of minor salivary glands, and neurofibroma [12,15]. Clinically, mesenchymal carcinoma of the minor salivary glands often presents as a benign tumor or with inflammation [1,3,15,16]. Most patients experience an asymptomatic swelling, and in many cases, a fluctuant, bluish, smooth-surfaced swelling appears, resembling a retention cyst [1,3,15,16].

For diagnosis, physical examination should be complemented with imaging tests such as computed tomography (CT) and magnetic resonance imaging (MRI), which are essential for staging salivary gland tumors [3,17]. Imaging features are nonspecific and vary with tumor grade: low-grade tumors tend to be well-defined masses, often within the parotid gland, whereas higher-grade tumors show local invasion and lymph node metastases. T2-weighted signal intensity and post-contrast images also correlate with tumor grade; more aggressive, highly cellular lesions tend to be hypointense on T2 and show necrosis on T2-weighted and post-contrast sequences [1]. Low-grade mucoepidermoid carcinomas typically have a pseudocystic appearance, especially on CT [1]. Radiologic assessment of salivary gland lesions in children is often complex because of the nonspecific nature of imaging [2]. In this case, the clinical presentation and fluctuating course initially pointed to a benign lesion, and the absence of significant findings on CT supported that impression (Figure 1).

From a histopathological standpoint, MECs are classified based on the number of cystic zones, degree of cellular atypia, relative counts of mucous, epidermoid, and intermediate cells, areas of necrosis, and involvement of bone, vessels, and nerves. This classification divides them into low-, intermediate-, and high-grade groups, as proposed by Healey et al. in 1970 [4,18]. Treatment decisions rely on histological grading.

Low-grade MECs tend to form cystic structures, exhibit minimal cellular atypia, and have a higher proportion of mucous cells; treatment involves surgical resection [4], as seen in this case. Because the lesion was found incidentally and initially treated as a mucocele, complete resection was performed; however, the margins were initially unclear. The histopathology report identified a low-grade MEC, prompting a PET scan to evaluate cervical involvement, which was negative. A second resection was then performed with margins in healthy tissue (Figure 3). Surgery for palatal MEC usually involves wide local excision followed by wound healing, as was done in our patient.

High-grade MECs consist of solid islands of squamous and intermediate cells, with considerable pleomorphism and mitotic activity. Intermediate-grade MECs exhibit characteristics of both grades mentioned above. Treatment for intermediate- and high-grade MECs involves oncologic resection with intraoperative rapid biopsy, with or without neck dissection. Key considerations for assessing the involved lymph node levels include clinical parameters such as the presence of lymphadenopathy on physical examination, imaging findings, tumor extent, bone involvement, and histological grade [4,14,15].

The 10-year overall survival rates for low-, intermediate-, and high-grade MECs are 90%, 70%, and 25%, respectively [19]. In low-grade MECs, metastatic disease is uncommon [3,12].

Therefore, our patient will be followed up for 10 years, with periodic visits to the clinical and surgical oncology and otolaryngology services.

## Conclusions

Malignant tumors of the minor salivary glands appear sporadically in children. MECs are the most common entity, although they are rare in the first decade of life and are more common in females; they can mimic benign lesions such as mucoceles. In this case, the need to maintain a high index of suspicion and to always submit resected tissue for histopathological examination is underscored. Early detection and appropriate multidisciplinary management guarantee an excellent prognosis.
